# Changes in Cerebral Oxidative Metabolism during Neonatal Seizures Following Hypoxic–Ischemic Brain Injury

**DOI:** 10.3389/fped.2016.00083

**Published:** 2016-08-10

**Authors:** Subhabrata Mitra, Gemma Bale, Sean Mathieson, Cristina Uria-Avellanal, Judith Meek, Ilias Tachtsidis, Nicola J. Robertson

**Affiliations:** ^1^Department of Neonatology, Institute for Women’s Health, University College London, London, UK; ^2^Department of Medical Physics and Biomedical Engineering, University College London, London, UK

**Keywords:** near-infrared spectroscopy, cytochrome-*c*-oxidase, hypoxic–ischemic encephalopathy, seizures, electroencephalography

## Abstract

Seizures are common following hypoxic–ischemic brain injury in newborn infants. Prolonged or recurrent seizures have been shown to exacerbate neuronal damage in the developing brain; however, the precise mechanism is not fully understood. Cytochrome-*c*-oxidase is responsible for more than 90% of ATP production inside mitochondria. Using a novel broadband near-infrared spectroscopy system, we measured the concentration changes in the oxidation state of cerebral cytochrome-*c*-oxidase (Δ[oxCCO]) and hemodynamics during recurrent neonatal seizures following hypoxic–ischemic encephalopathy in a newborn infant. A rapid increase in Δ[oxCCO] was noted at the onset of seizures along with a rise in the baseline of amplitude-integrated electroencephalogram. Cerebral oxygenation and cerebral blood volume fell just prior to the seizure onset but recovered rapidly during seizures. Δ[oxCCO] during seizures correlated with changes in mean electroencephalogram voltage indicating an increase in neuronal activation and energy demand. The progressive decline in the Δ[oxCCO] baseline during seizures suggests a progressive decrease of mitochondrial oxidative metabolism.

## Introduction

Seizures occur in ~75% of infants with hypoxic–ischemic encephalopathy (HIE) (1). Animal studies have indicated that prolonged and frequent seizures in the developing brain result in long-term neurological sequelae (2). Clinical studies also suggest that neonatal seizures are independently associated with further neuronal injury (3) and poor outcome (4, 5); however, the mechanisms for these harmful effects are not clear.

Mitochondrial metabolism is closely related to neuronal activity. Studies using phosphorus-31 magnetic resonance spectroscopy (^31^P MRS) have revealed that high-energy phosphates decrease by one-third and mitochondrial oxidative phosphorylation increases by 45% during neonatal seizures (5), indicating a depleted cerebral energy state during seizures.

Near-infrared spectroscopy (NIRS) measures concentration changes in oxygenated (Δ[HbO_2_]) and deoxygenated hemoglobin (Δ[HHb]) which can be used to derive changes in total hemoglobin (Δ[HbT] = Δ[HbO_2_] + Δ[HHb]) and hemoglobin difference (Δ[HbD] = Δ[HbO_2_] − Δ[HHb]). Changes in [HbT] and [HbD] reflect changes in cerebral blood volume and brain oxygenation, respectively. Broadband NIRS can measure the changes in the oxidation state of cytochrome-*c*-oxidase (Δ[oxCCO]), which indicate fluxes in mitochondrial oxidative metabolism. Previous NIRS studies during neonatal seizures have described cerebral hemodynamics and oxygenation (6–9), but changes in cerebral mitochondrial [oxCCO] have not yet been assessed in humans during seizures.

Cytochrome-*c*-oxidase is the terminal electron acceptor in the electron transport chain (ETC). It plays a crucial role in mitochondrial oxidative metabolism and ATP synthesis. CCO contains four active metal redox centers; one of them, the CuA is a dominant near infrared (NIR) chromophore and the primary contributor for the NIR spectral signature (10). As the total concentration of CCO is assumed constant, the changes in oxCCO concentration indicate changes in CCO oxidation state in cerebral tissue, representing the status of mitochondrial oxidative metabolism. Several previous studies have indicated that cytochrome-*c*-oxidase has the potential to monitor mitochondrial activity and cerebral metabolism (11, 12). Our group has previously described a significant association between the oxidation state of cerebral mitochondrial cytochrome-*c*-oxidase (Δ[oxCCO]) and ^31^P metabolite peak-area ratios during and after transient cerebral hypoxia-ischemia (HI) in newborn piglets (13).

Broadband NIRS can accurately resolve spectral changes due to oxCCO without cross talk from the hemoglobin chromophores. We have recently developed a new broadband NIRS system, which is capable of measuring Δ[oxCCO] as well as Δ[HbO_2_], and Δ[HHb] in the neonatal brain (14). In this report, we evaluate the changes in cerebral mitochondrial oxidative metabolism synchronously with changes in cerebral oxygenation, hemodynamics, and electroencephalogram (EEG) during neonatal seizures following hypoxic–ischemic brain injury.

## Background

### Subject

**Figure 1:**
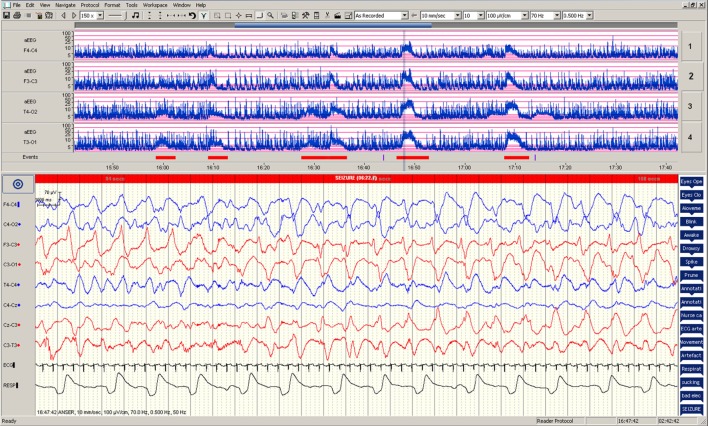
**Electrographic changes during the seizures**. Amplitude-integrated electroencephalogram (aEEG) recording from F4–C4, F3–C3, T4–O2, and T3–O1 are presented in the upper panel with five seizure episodes marked in red. Electroencephalographic (EEG) changes during the fourth seizure are presented in the lower panel.

Ethical approval from the local Research Ethics Center and informed parental consent were obtained. Recurrent seizures were studied in a term infant (born at 38 + 2 weeks, birth weight 3034 g), delivered by emergency Cesarean section after placental abruption. A neurological examination with Sarnat staging indicated moderate encephalopathy. The infant received therapeutic hypothermia (body temperature reduced to 33.5°C and was maintained same for 72 h) as part of the standard neuroprotective strategy following HIE. Rewarming was commenced following this with servo-controlled increase in body temperature of 0.5°C over every 2 h. During rewarming (at ~80 h of age), the baby developed recurrent seizures (only electrographic seizures were noted, no clinical changes seen) (Figure 1), which prompted further hypothermic treatment (temperature was reduced by 1°C and was maintained same for 4 h) followed by slow rewarming (increase of 0.5°C over every 4 h) to 37°C. A bolus dose of phenobarbitone was commenced after the second seizure, and the infant remained seizure-free after this episode of five recurrent seizures. The infant was ventilated for first 6 days of life and needed inotropic support on day 1. MRI of brain on day 6 revealed abnormal appearances to the basal ganglia, thalami, and posterior limb of internal capsule with some exaggerated low signal intensity around lateral thalamic nucleus on diffusion map. At the time of discharge, the infant was noted to be slightly hypotonic in both upper and lower limbs but had good sucking reflex and established breastfeeding.

### Multimodal Data Collection

Multimodal data were collected from broadband NIRS, EEG, and systemic monitors over 90 min. NIRS probes placed on both sides of the forehead, collected data at 1 Hz using a 2.5 cm optode distance. Both NIRS channels measured similar changes over the left and right side of the forehead.

A 10-channel neonatal EEG was acquired on a Nicolet EEG monitor (Natus Medical, USA), and amplitude-integrated EEG (aEEG) trends were derived. Mean aEEG was calculated from the mean of the upper and lower values of the aEEG band. Seizure onset and offset times were annotated from EEG. Systemic data from the Intellivue Monitors (Philips Healthcare, UK) were collected using ixTrend software (ixellence, Germany), down-sampled and interpolated to the broadband NIRS data timeframe (1 Hz).

### Data Analysis

Relationships between Δ[oxCCO], Δ[HbD], Δ[HbT], and mean aEEG during seizures were analyzed by principal component analysis (PCA). Results are presented as means ± SD unless otherwise indicated. Statistical analyses were performed using JMP 11 (SAS Institute, USA) and Prism 6 (GraphPad, USA).

### Results

**Figure 2:**
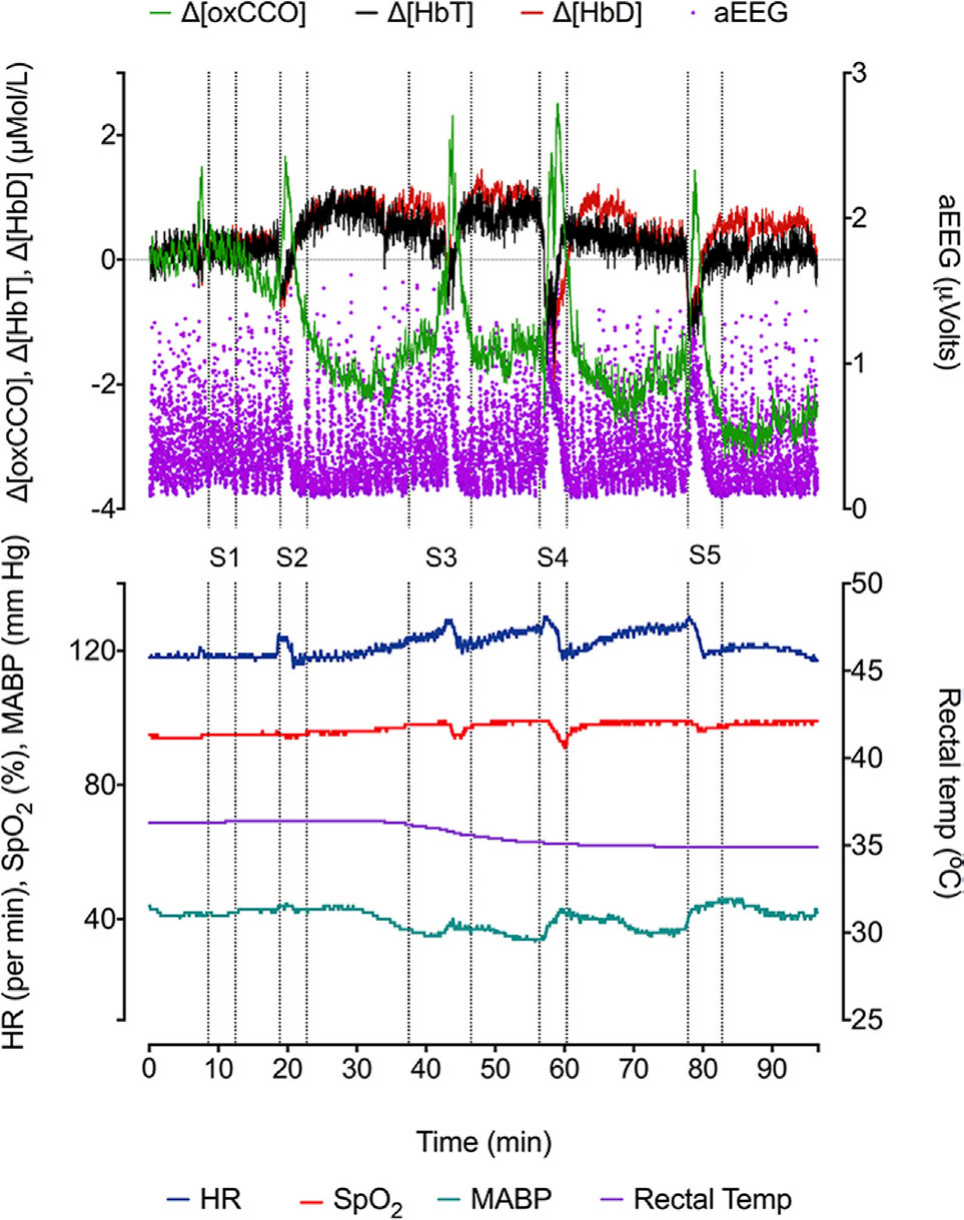
**Changes in NIRS variables (Δ[HbD], Δ[HbT], and Δ[oxCCO]), aEEG (F3–C3), and systemic parameters [heart rate (HR), peripheral oxygen saturation (SpO_2_), mean blood pressure (BP mean), and rectal temperature (°C)] during five recurrent seizure episodes (S1, S2, S3, S4, and S5)**.

**Figure 3:**
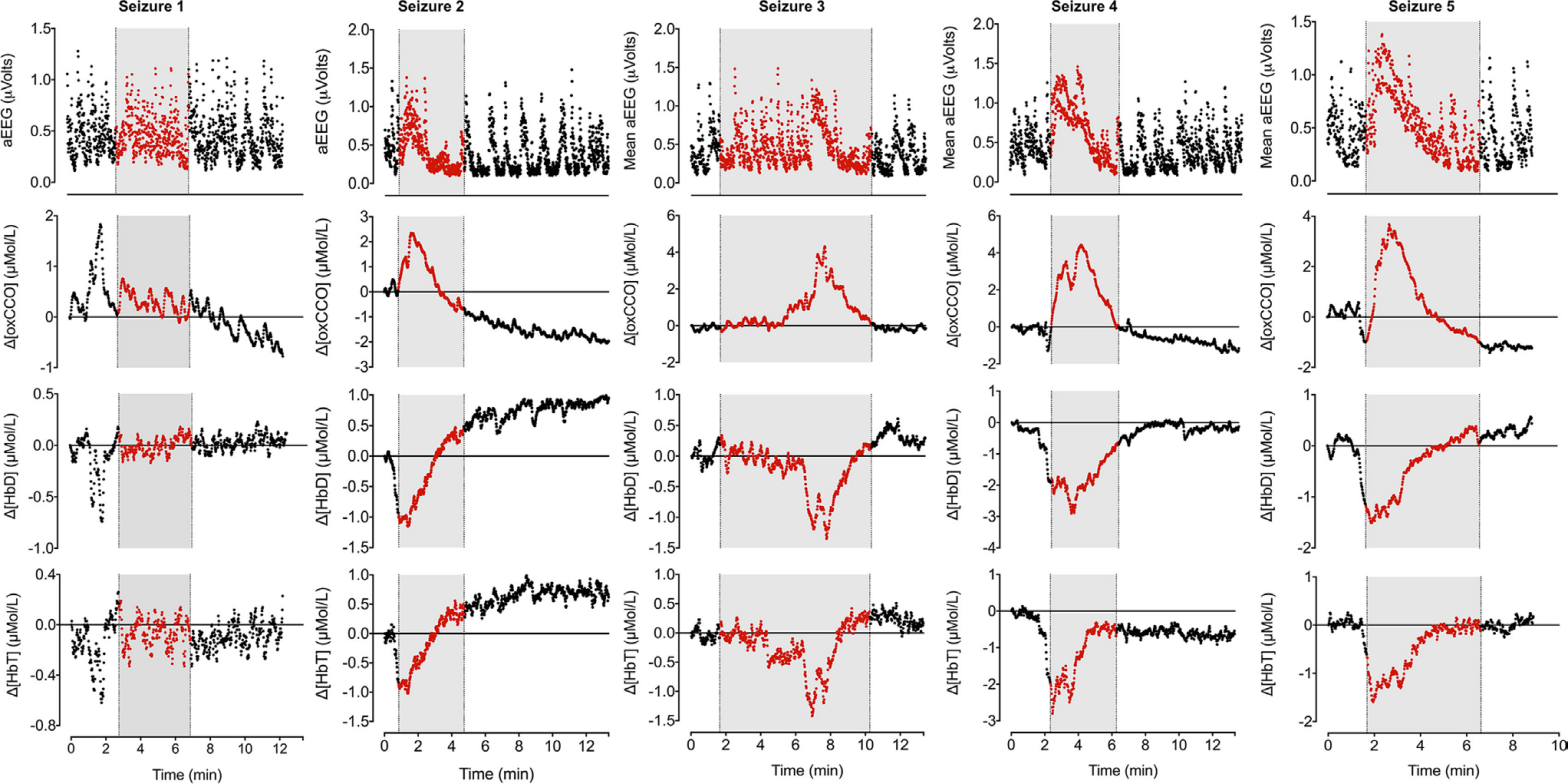
**Changes in mean aEEG, Δ[oxCCO], Δ[HbD], and Δ[HbT] during seizures**. Mean aEEG was calculated from F3–C3 channel. NIRS variables have been adjusted to start from 0 on each occasion to reflect the true changes. Periods of electrographic seizures on EEG have been marked in gray and each variable has been marked in red during the seizures. Seizures 2–5 show similar pattern of changes in aEEG, Δ[oxCCO], Δ[HbD], and Δ[HbT]. Δ[oxCCO] increases along with an increase in mean aEEG baseline followed by a drop after peak electrical activity while Δ[HbD] and Δ[HbT] drop early in the preictal period (seizures 2, 4, and 5) and subsequently recover toward baseline. Δ[oxCCO] drops initially in seizures 4 and 5 along with Δ[HbD] and Δ[HbT] but increases with the rise of mean aEEG baseline. Changes in NIRS variables in seizure 1 was similar to other seizures but were noted prior to the onset of electrographic seizure. The first two seizures originated from left posterior hemisphere; the rest were generalized seizures.

**Figure 4:**
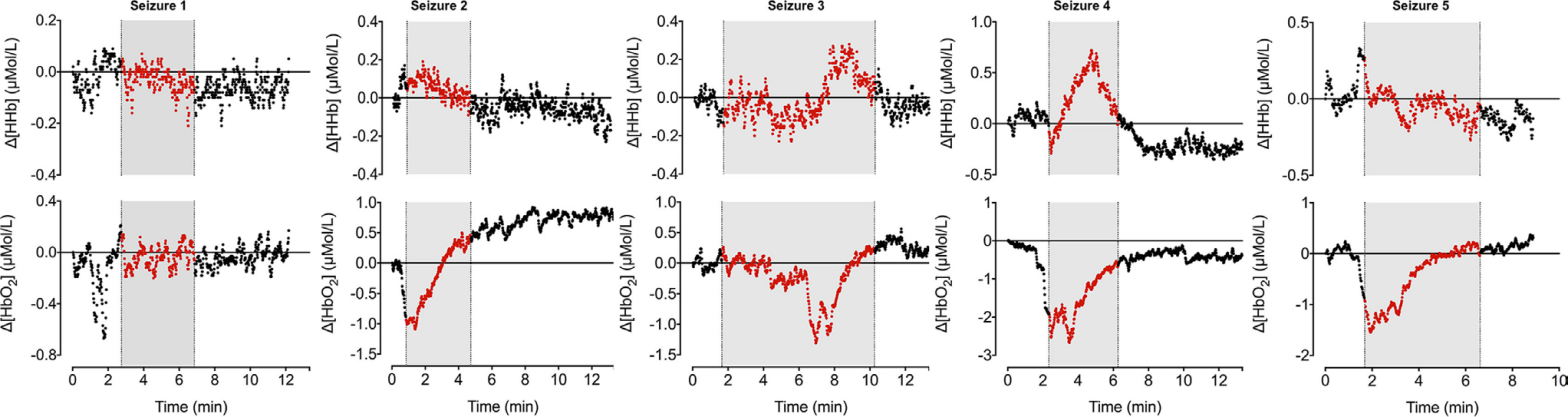
**Changes in oxy- and deoxy hemoglobin ([HbO_2_] and [HHb]) during seizures**. [HHb] increases and decreases during ictal period while [HbO_2_] follows opposite trend.

During the 90-min data collection, 5 subclinical seizures were recorded (Figures 1 and 2). Mean seizure duration was 5.03 min (3.53–8.36 min). At the start of each seizure on the aEEG (indicated by a rise in the baseline of the aEEG), the Δ[oxCCO] increased by 3.30 ± 1 μMol/L. Following the peak of the aEEG activity, the Δ[oxCCO] started to fall and continued to fall even after the end of each seizure to a progressively lower baseline, which at the end of the study was at −4.19 μMol/L (Figure 2). Δ[HbT] and Δ[HbD] both decreased initially by 1.51 ± 0.77 and 1.50 ± 0.69 μMol/L, respectively, but soon returned toward baseline after the peak of the aEEG activity (Figure 3). Δ[HHb] increased and Δ[HbO_2_] fell during seizures, but returned toward the baseline subsequently (Figure 4). Heart rate (HR) and mean arterial blood pressure (MABP) increased during seizures by 5 ± 1.1 beats/min and 5 ± 2.9 mm Hg, respectively, while peripheral arterial oxygen saturation (SpO_2_) dropped by 3.2 ± 2.8%. Rectal temperature during the study was reduced by 1°C (Figure 2).

**Figure 5:**
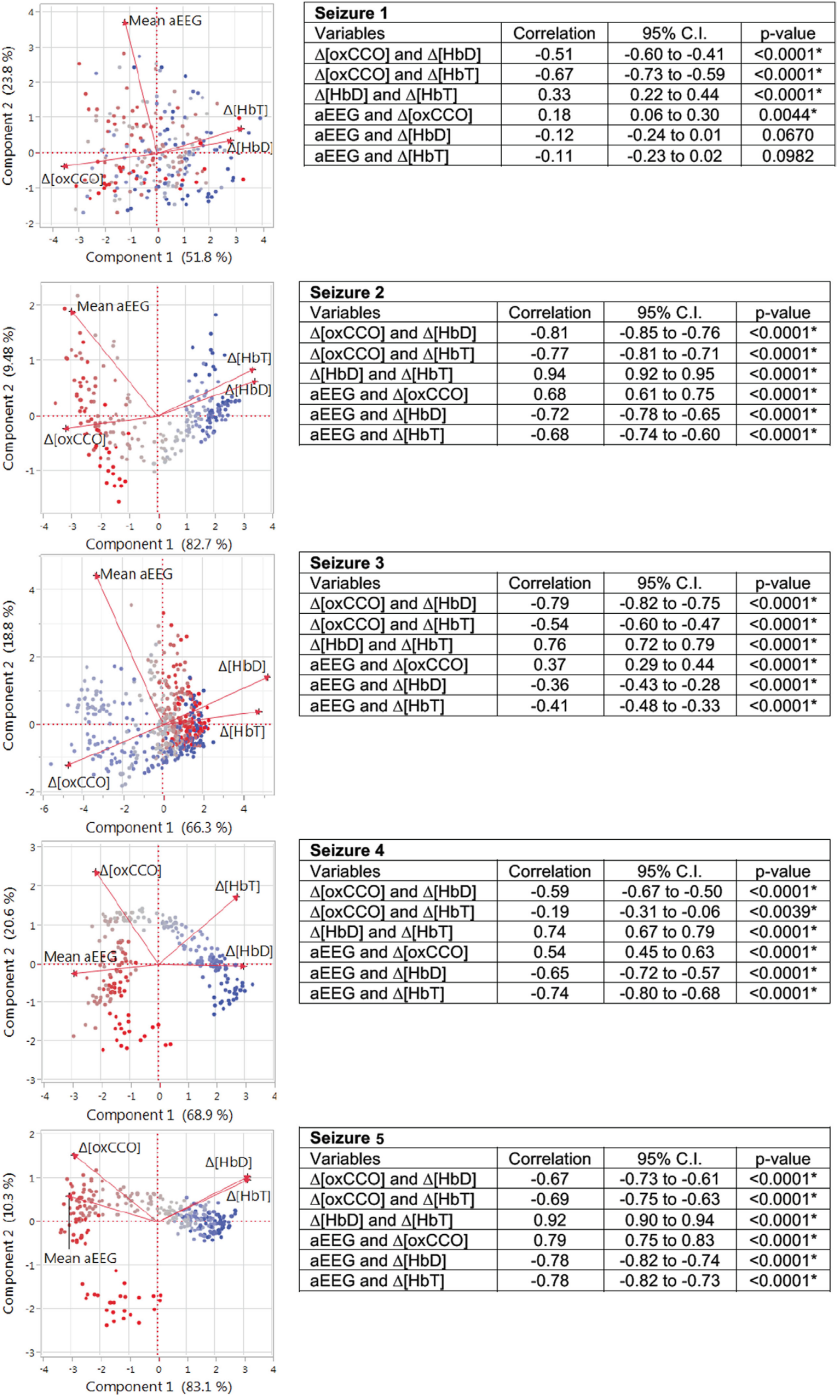
**Matrix of correlation coefficients and corresponding data table for each seizure were analyzed with principal component analysis**. Pairwise correlations between the variables with 95% confidence interval (CI) are presented in the table with the significance level. Matrix of correlation coefficients summarizes the strength of the co-relationships between each pair of variables and colors the strength of each correlation on a scale from red (+1 strongest positive correlation) to blue (−1 strongest negative correlation). The closer it is toward 0, the weaker the correlation and color. Mean aEEG was significantly correlated positively with Δ[oxCCO]. Both Δ[HbD] and Δ[HbT] were negatively correlated with mean aEEG and Δ[oxCCO].

Broadband NIRS measured Δ[oxCCO] was closely related to the mean aEEG changes (PCA correlation coefficient 0.51 ± 0.22 during all seizures, *p* < 0.005) (Figure 3). Both Δ[HbD] and Δ[HbT] were negatively correlated with mean aEEG (−0.53 ± 0.25, *p* < 0.0001 and −0.54 ± 0.25, *p* < 0.0001, respectively) and Δ[oxCCO] (PCA correlation coefficient −0.67 ± 0.12, *p* < 0.0001 and −0.57 ± 0.20, *p* < 0.004, respectively) (Figure 5).

## Discussion

This is the first report of Δ[oxCCO] fluxes during recurrent seizures in the neonatal brain following perinatal hypoxic–ischemic injury. These fluxes are described relative to changes in cerebral oxygenation, hemodynamics, and electrophysiology. Neuronal energy demand rapidly increased at the onset of seizures reflected by a rapid increase in the mean aEEG activity coinciding with a rise in Δ[oxCCO]. These changes in [oxCCO] occurred even when cerebral tissue oxygenation and hemodynamics were compromised (both Δ[HbD] and Δ[HbT] started to fall before the onset of seizures). After the peak of the seizure activity, energy consumption decreased and Δ[oxCCO] returned toward and below baseline.

Preclinical studies show that broadband NIRS measured CCO signal follows the same trajectory and correlates with high-energy phosphates during primary and secondary energy failure following hypoxic–ischemic brain injury, indicating its ability to represent the changes in mitochondrial energy state (13, 15). High-energy phosphate stores have also been shown to decline during seizures in both clinical and preclinical studies (5, 16). Our observed increase in mitochondrial oxidative metabolism during neonatal seizures concurs with these findings and the progressive decrease in [oxCCO] baseline with recurrent seizures in our study indicated a decrease in mitochondrial oxidative metabolism. As cerebral glycogen stores and NADH decline during seizures (16), this fall in substrate supply further leads to the increase in the oxidation of cytochrome-*c*-oxidase (17).

Cerebral oxygenation fell rapidly before the onset of electrographic seizures but soon recovered in parallel with cerebral blood volume. Both parameters continued to increase during the ictal period. Although the cerebral blood volume and oxygenation stabilized, the Δ[oxCCO] continued to drop in the postictal period. A similar mismatch between cerebral hemodynamics and metabolism during post-asphyxial seizures has been described in near-term fetal sheep (18). Frontal preictal hemodynamics (fall in Δ[HbD] and Δ[HbT] during seizures 2, 4, and 5) and metabolic changes (fall in Δ[oxCCO] during seizures 4 and 5) were noted prior to the onset of seizures (Figure 3). Preictal frontal hemodynamic changes have been previously described in a neonate (9) and in adults (19). These preictal changes indicate an imminent electrographic seizure. The early preictal drop in Δ[oxCCO] coinciding with a drop in cerebral oxygenation during seizures 4 and 5 indicates a more linear oxygen dependency of Δ[oxCCO] [compared with an initial metabolic buffering period noted during transient anoxia in newborn piglet brain (20)]. This becomes more evident with decreasing mitochondrial energy production following repeated seizures. Oxygenation along with changes in substrate supply and the energy demand are the most important physiological stimuli to influence the redox state of CCO within the ETC. Availability of oxygen relates to oxidation state of CCO in an asymptotic fashion (20, 21).

Our clinical data complement and extend the previous preclinical studies, which have shown intraneuronal depletion of ATP and increase of ADP (22), decrease in cortical tissue pH (16), increased glycolytic flux (16), increased cerebral oxygen consumption, and oxidation of intramitochondrial NADH during seizures (23).

We are not able to comment whether [oxCCO] baseline would have returned toward baseline after seizure cessation as our recording stopped at 90 min. However, our observed Δ[oxCCO] baseline drift during the study could be related to increased adenosine concentrations, resulting in suppression of mitochondrial metabolism. Excessive neuronal activation, as occurs during a seizure, leads to neuronal release of adenosine that acts on synapses (24, 25) and terminates seizures (26). Interestingly, the EEG background remained suppressed for another 30 min after we stopped NIRS monitoring. A clinical decision was taken after the second seizure to stop rewarming, lower the body temperature by 1°C, and commence a bolus dose of phenobarbitone. Ictal changes in cerebral metabolism and hemodynamics followed similar pattern before and after these changes. Ventilatory oxygen delivery and transcutaneous CO_2_ readings remained stable during the study.

## Concluding Remarks

We present a set of novel bedside observations related to brain metabolism during seizures in the newborn brain after perinatal hypoxic–ischemic injury. A rapid increase in Δ[oxCCO], a non-invasive real-time measurement of mitochondrial oxidative metabolism, at the onset of seizures correlated with changes in mean EEG voltage indicating an increase in neuronal activation and energy demand. The progressive fall in the Δ[oxCCO] baseline during repeated seizures indicated a decrease in mitochondrial oxidative metabolism, which could explain the exacerbation of brain injury after repeated or prolonged seizures. However, the interpretation of these measurements is complex, and it is unclear to what extent such changes contribute to long-term neurodevelopmental outcome.

## Author Contributions

S. Mitra, IT, and NR wrote the first draft of the manuscript. S. Mitra, IT, NR, GB, S. Mathieson, CU-A, and JM are responsible for the reported research and have participated in the concept and design, analysis and interpretation of data, drafting and revising the manuscript.

## Conflict of Interest Statement

All authors confirm that there are no financial or other relationships that might lead to a perceived conflict of interest.

## Abbreviation

oxCCO, cytochrome-*c*-oxidase oxidation state.
